# The Effect of Anemia and the Goal of Optimal HbA1c Control in Diabetes and Non-Diabetes

**DOI:** 10.7759/cureus.8431

**Published:** 2020-06-03

**Authors:** Prakash C Katwal, Srood Jirjees, Zin Mar Htun, Israa Aldawudi, Safeera Khan

**Affiliations:** 1 Internal Medicine, California Institute of Behavioral Neurosciences and Psychology, Fairfield, USA; 2 Neurology, California Institute of Behavioral Neurosciences and Psychology, Fairfield, USA; 3 Radiology, California Institute of Behavioral Neurosciences and Psychology, Fairfield, USA

**Keywords:** hba1c, diabetes, hemoglobin, anemia, iron deficiency anemia (ida)

## Abstract

Hemoglobin A1c (HbA1c) is the gold standard for the diagnosis of diabetes; however, many clinical conditions affect the HbA1c level, including anemia. And, the most common causes of anemia worldwide include iron deficiency anemia (IDA). We performed a systematic search using different combinations of MeSH words from the electronic database for the last 10 years (2011 to 2020). Articles included in the study were observational, randomized controlled trial (RCT), and review/systematic review. A total of 18 articles were included in the study. The majority of the studies showed the association between hemoglobin (Hb) and HbA1c. Large-scale studies showed that the HbA1c level increases in IDA and some studies showed its correction after the treatment with oral iron supplementation. Our study indicates the need for screening for anemia in patients before commencing the treatment of diabetes diagnosed via the HbA1c level. Furthermore, anemia should be corrected before setting the treatment goal of optimal HbA1c control, especially when the level is in the diagnostic threshold. Also, the purpose of strict HbA1c control is not recommended in the anemic patient before it is corrected. However, further large-scale interventional studies are needed to know precisely the goal of optimal HbA1c control in diabetic and non-diabetic individuals.

## Introduction and background

Anemia is defined as a reduction in the oxygen-carrying capacity of blood and measured by the cut-off value of Hb level <12.0 mg/dl in adult non-pregnant women and <13.0 mg/dl in adult men [1]. The prevalence of anemia is 32.9% worldwide, and among many causes of anemia, iron deficiency anemia (IDA) is the most common cause [2]. 

Diabetes mellitus (DM) is a clinical syndrome occurring due to an absolute or relative deficiency of insulin, leading to abnormal carbohydrate metabolism. It is manifested clinically as hyperglycemia and is broadly classified as Type I and Type II DM, of which >85% disease burden is due to Type II [3]. The diagnosis of DM is made on the basis of fasting, two-hour postprandial, random blood sugar, and HbA1c. In euglycemic person HbA1c is < 5.5%, impaired glucose tolerance 5.6%-6.5% and >6.5% is considered diabetic [4].

Hemolytic anemia leads to the reduced lifespan of red blood cells (RBC), and IDA prolongs it; as a result, there occurs the discrepancy in hemoglobin A1c (HbA1c) [5]. HbA1c is the product of the non-enzymatic chemical reaction of Hb with glucose. It reflects the average amount of blood glucose in the past two to three months [6]. HbA1c is an important tool to assess glycaemic control, and its regular monitoring and strict control reduce the onset and progression of wide varieties of diabetic-related complications [7].

Furthermore, HbA1c is one of the gold standard tests’ in the diagnosis of DM and monitoring of long-term blood glucose levels [8], in anemia because these causes can lead to erroneous increase or decrease in HbA1c leading to misinterpretation of the disease condition. So, the significance of Hb value in serial monitoring of HbA1c and the cut-off value of Hb below which HbA1c cannot be considered as important diagnostic and monitoring tools in diabetes is still a mystery [6].

There has been a controversy for the use of HbA1c as a diagnostic tool in anemia. As a result, we aim to find the effect of anemia and the goal of optimal HbA1c control in diabetes and non-diabetes from the available evidence.

## Review

Methods

We performed a systematic search on PubMed, PMC, and Google scholar and searched for the studies published in the last 10 years. Keywords used in different combinations were: “HbA1c”, “Diabetes and HbA1c”, “Diabetes and Anemia,” “HbA1c and Anemia and Diabetes,” as shown in Table 1. All articles published in English between this period were considered for review. Search engines like “Google,” “Medscape,” “UpToDate” were also used to find the related articles. Reference lists of all the articles were manually checked to identify studies not found through electronic searching. Quality checks were not performed for all studies.

Approval of an ethics committee was not required.

**Table 1:** Electronic search results HbA1c, hemoglobin A1c

Keyword/MeSH	Date	Databased used	No of paper/result
HbA1C	05/03/2020	PubMed	53,071
Diabetes and HbA1c	05/03/2020	PubMed	48,668
Diabetes and Anemia	05/03/2020	PubMed	6,540
HbA1c and Anemia and Diabetes	05/03/2020	PubMed	380
HbA1c and Anemia and Diabetes	05/03/2020	PMC	4,022
HbA1c and Anemia and Diabetes	05/03/2020	Google Scholar	20,400

Inclusion and exclusion criteria

All the articles relevant to the topic were searched in different databases. Only articles published in English medium with full text between 2011 to 2020 and articles from all the geographical locations were included. All types of studies were included except animal studies.

Result

**Figure 1:**
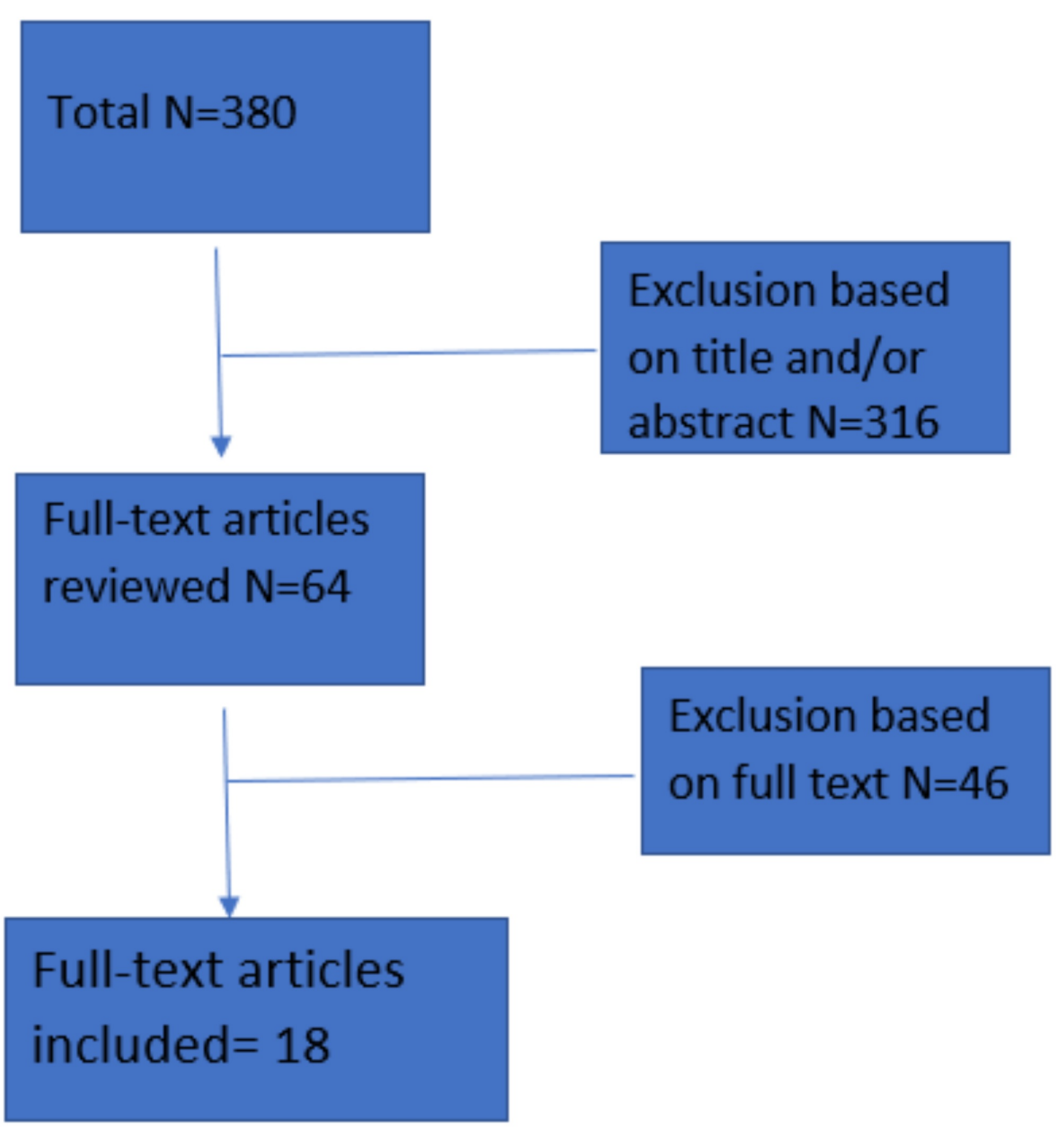
Flow chart of the search strategy N, number of articles

The database searches identified 380 related articles out of which 316 articles were excluded based on title and/or abstract, which were not relevant to our study. Out of the remaining 64 full-text articles, 46 were excluded after further reading, and 18 articles were approved for the study. The main reason for exclusion was incomplete data and/or not meeting the inclusion criteria. Included articles had at least one measurement of HbA1c and hemoglobin by any method; however, this rule was not applied for the review articles. No additional articles were identified via reviews of reference lists (Figure 1)

Discussion

Various studies have been conducted to gauze the relationship between anemia and HbA1c. More studies were directed towards IDA. The effect of IDA on the HbA1c level was first studied by two American researchers Horton and Huisman, in 1965 [9]. Ongoing studies have been done since then, which are out of the scope of this review article and can be found in various journals. We have tried to review related articles from 2011 to till date, which are depicted in Table 2 and Table 3.

**Table 2:** Observational/RCT studies IDA, iron deficiency anemia; HbA1c, hemoglobin A1c; Hb, hemoglobin; LID, latent iron deficiency; RCT, randomized controlled trials; HPLC, high-performance liquid chromatography

Source	Sample size	Age group	Study type	HbA1c (%)	Hb level	Result
Solomon et al. 2019 [10]	174	>18 years	Cross-Sectional	IDA: 6.18+/-1.57 Non-IDA: 7.74+/-1.81 (P<0.05)	IDA: 9.97+/-2.04 Non-IDA: 15.17+/-1.21 (P<0.05)	HbA1c is significantly lower in diabetic with IDA compared with non-IDA (P<0.05)
Kalairajan et al. 2019 [11]	Case: 120; Control: 120	18-60 years	Prospective Interventional	IDA: 4.62+/-0.30 Non-IDA: 5.45+/-0.28 (P<0.001). After correction: HbA1c: 5.82+/-0.32 (P<0.001)	IDA: 6.8+/-1.08 Non-IDA: 13.4+/-0.35 (P<0.001). After correction: 12.7+/-0.44 (P<0.001)	The significant correlation observed between Hb and HbA1c level (coefficient of correlation: 0.26; P<0.01)
Urrechaga 2018 [15]	661	>18 years	Cross-Sectional	Female >50 years: 7.0+/-1.5 <50 years: 6/3+/-1.3. Male: >50 years: 7.0+/-1.6 <50 years: 6.7+/-1.6	LID: >120g/L, IDA: male: <130 g/L, female: <120 g/L	A positive correlation between HbA1c and IDA
Madhu et al. 2017 [16]	122	20-70 years	Case-Control	Case: 5.5+/- 0.7. Control: 4.9 +/- 0.5 (P<0.001)	Case: 73.9+/-12.2. Control: 134.3+/-13.2 (P<0.001)	Significantly higher HbA1c in IDA P <0.001 and significant improvement in HbA1c level after oral iron supplementation
Alsayegh et al. 2017 [12]	1580	18-71 years	Cross-Sectional	>7 anemia (80.7%), non-anemia (80.4%) <7. Anemia (19.3%), non-anemia (19.6%)	Anemia: male: <130 g/L, female: <120 g/L	Higher prevalence of anemia in the diabetic patient (P<0.001). Furthermore, diabetic peripheral neuropathy and diabetic foot were commonly associated with anemia. However, there was no association between HbA1c and Hb (P=0.887)
Inada and Koga 2017 [17]	35	Non-IDA 59.0+/-7.8. IDA: 59.1+/-2.2 years	Case-Control	IDA: 6.2+/-0.4%. Non-IDA: 5.7+/-0.3 (P=0.003)	Without anemia: 139+/-0.8. With IDA: 11.1+/-0.9 (P<0.0001)	HbA1c level is higher in gastrectomized subjects with IDA than non-IDA (P=0.003)
Esfahani et al. 2017 [18]	90 (45 cases, 45 control)	18-65 years	RCT	Pre: case 7.59+/-1.16, control: 7.40+/-1.01. Post: case: 6.80+/-0.85, control: 7.14+/-0.95 (P<0.001)	Pre: case: 11.52+/-0.86, control: 11.3+/-0.73. Post: case: 13.71+/-1.37, control: 11.6+/-1.24 (P<0.001)	Significant improvement in the HbA1c level after treatment with iron therapy in anemia patient with IDA and Type II diabetes
Silva et al. 2016 [19]	122	18-77 years	Case-Control	Anemia: 5.6+/-0.4 (HPLC method) 5.7+/-0.4 (Immunoturbidimetry Method). No Anemia: 5.3+/-0.4(HPLC Method) 5.3+/-0.3 (Immunoturbidimetry Method) (P<0.001)	Mild: Male: 11-13 mg/dl. Female: 11-12 mg/dl. Moderate: 8-11 mg/dl. Severe: <8 mg/dl	IDA affects HbA1c value, which depends on the severity of anemia. In cases of mild anemia, there is a minimal effect in HbA1c and can be used as a diagnostic tool for diabetes
Hong et al. 2015 [20]	10665	>19 years	Cross-Sectional	No Anemia: 5.59+/ 0.01. Non-IDA: 5.44+/-0.03. IDA: 5.70+/-0.02 (P<0.001)	No anemia: 14.4+/-0.1. Non-IDA: 12.3+/-0.1. IDA: 11.5+/-0.1 (P<0.001)	No significant difference in the HbA1c level between IDA and non-IDA. However, in euglycemic and prediabetic, HbA1c is significantly higher in IDA compared to non-IDA
Christy et al. 2014 [21]	120	>18 years	Case-Control	IDA: 6.87+/-1.4, Non-IDA: 5.65+/-0.69	Low Hb: Male <12 gm%, female <11 gm%	A positive correlation between IDA and increase HbA1c level, especially in controlled diabetes women and individuals with fasting plasma glucose 100-126 mg/dl
Shanthi et al. 2013 [22]	Case: 50. Control:50	43.52+/-7.79	Case-Control	In IDA: 7.6+/-0.5%. Non-IDA: 5.5+/-0.8% (P<0.001)	IDA: 10.6+/-1.4. Non-IDA: 13.4+/-0.96%	The author concluded that there is a significant positive correlation between HbA1c and IDA. So, it is important to screen for IDA before starting the treatment for diabetes
Ford et al. 2011 [14]	8296	>=20 years	Cross-Sectional	Mean HbA1c 5.28% (in Hb<100g/L) & 5.72% (In Hb >170g/L). Adjusted mean HbA1c, in IDA: 5.56%. Non-IDA: 5.46% (P=0.095)	Prevalence of anemia IDA: 2.3+/-0.2% Non-IDA: 3.2+/-0.3%. Normal Hb with IDA: 4.4+/-0.3%. Normal Hb and normal Iron: 90.1+/-0.4%	A significant positive correlation between Hb concentration and HbA1c concentration. Furthermore, caution should be made in diagnosing diabetes in patients with anemia who are close to the diagnostic threshold (6.5% & 5.7%) and need another method or retesting for diagnosis
Villar et al. 2011 [13]	89	18-80 years	RCT	Sub-normal: 7.5 +/-1.3. Normal: 7.6 +/-1.4 (P=0.73)	Sub-normal Hb: 110-129. Normal: 130-149 gm/L	HbA1c did not vary significantly with anemia correction in chronic kidney disease (P>0.05)

**Table 3:** Review articles and their conclusions HbA1c, hemoglobin A1c; IDA, iron deficiency anemia

Source	Study type	Result
Guo et al. 2019 [6]	Review	Effects of IDA on HbA1c are multifactorial and multi-dimensional however
Naqash and Bader 2018 [23]	Review	HbA1c level is dependent on erythrocyte turnover. So, in IDA HbA1c level increased. However, the clinical data is insufficient
English et al. 2015 [7]	Systematic Review	In IDA HbA1c level falsely increase while in non-IDA, it may decrease
Ahmad and Rafat 2013 [24]	Review of Study	People who are in the diagnostic threshold of diabetes, another method of diagnostic modalities, are required to diagnose in anemia patients
Weykamp 2013 [5]	Review	In anemia due to chronic disease, HbA1c is low due to decreased RBC survival, but in IDA, it is spuriously high

In 2019, Solomon et al. showed mean Hb, hematocrit, and red cell indices were low as expected in the IDA group [10]. His study revealed that this group had a much lower HbA1c level than the non-IDA group. Therefore, monitoring the diabetic patient with only the HbA1c level should be done with caution. This finding was consistent with an interventional study among 120 subjects by Kalairajan et al. in 2019 [11]. It showed that IDA leads to a decrease in HbA1c level with a subsequent rise in HbA1c level with its correction. This showed a positive correlation between Hb and HbA1c in IDA before treatment; however, no correlation was observed after correction of anemia by oral iron supplementation for three months. The author in this study acclaims that this was one of the few articles done in the Indian subcontinent where the cause of IDA is nutritional. They also claim that the study was unique and encompasses a wide variety of race and ethnic groups and people of low socio-economic status in that location.

These two studies were contradictory to other studies. As other studies have either failed to find the association between two or the association was reversed. An observational study conducted by Alsayegh et al. in 2017 showed a high prevalence of anemia in diabetes patients (female>male) predominantly more in people with diabetes complications [12]. However, it showed no association between HbA1c and anemia in both males and females. The author further added the need for early diagnosis and prompt treatment of anemia in diabetes patients as it was associated with grave complications of diabetes. The previously published study also supported this finding. The RCT by Villar et al. in patients with Type II diabetes and chronic kidney disease divided the subjects into sub-normal Hb (110-129 g/L) and normal Hb (130-149 g/L) were given iron and/or erythropoietin stimulating agent (ESA) for correction of anemia for 6,12 18, 24 months and HbA1c did not vary significantly (P = 0.73) with correction of anemia [13]. This diversion from the finding in the literature could be accredited to the type of anemia being non-IDA.

Ford et al. had a cross-sectional study of 8,296 patients and found a significant positive correlation between Hb concentration and HbA1c concentration [14]. However, the HbA1c was found to be higher in IDA patients, and HbA1c concentration in IDA patients was similar to non-IDA patients. This slight variation in the finding can be attributed to the positive association between hemoglobin and HbA1c being counter-balanced by a negative association between HbA1c concentration and iron profile. Furthermore, the author has warned that caution should be made in diagnosing diabetes in patients with anemia who are close to the diagnostic threshold and need another method or retesting for diagnosis.

 

The majority of findings we observed were contradictory to the above findings. A study by Urrechaga in 2018 divided the Type II diabetes patient according to iron status as normal, latent iron deficiency (LID) and IDA and the result showed a non-homogenous increase in the HbA1c values in both latent and apparent iron deficiency anemia in both genders and the age groups above and below 50 years regardless of fasting plasma glucose [15]. The finding of this study was comparable with the previous study by Shanthi et al. [22]. The authors further recommend screening for IDA before commencing treatment in diabetes. Similarly, a study conducted a year before by Madhu et al. in 122 patients also showed comparable findings [16]. Besides, it also showed that in such subjects, the HbA1c level dropped after oral iron supplementation for three months from 5.51+/-0.696 to post-treatment 5.044+/-0.603 with P-value <0.001. Seventy percent of IDA patients of this study were prediabetic, who became euglycemic after iron therapy. This study has similar findings with previous studies by Esfahani et al. [18]. Indirectly this study supports the suggestion of the large survey of Ford et al. to take caution in diagnosing diabetes in IDA patients with threshold HbA1c [14].

Hong et al. have tried to find out the significance of HbA1c as a screening tool in diabetes and prediabetes cases with IDA [20]. The author had compared no anemia, non-IDA, and IDA, and found HbA1c as 5.59+/-0.01, 5.44+/-0.03, and 5.70+/-0.02, respectively (P<0.001). They have further added that the presence of IDA affected the HbA1c distribution >=5.7% (the cut-off value for impaired glucose tolerance, IGT) and >=6.1% (Korean cut-off value for diabetes) but not in >=6.5% (WHO cut-off value for diabetes). So, IDA can significantly increase in the HbA1c level in the people with prediabetic glycaemic status as well as in normal glycaemic status but does not affect fasting plasma glucose and HbA1c level in diabetic people. This finding again supports the finding by Ford et al. and Madhu et al. that the HbA1c level in the diagnostic threshold is affected by IDA [14,16]. Similarly, a case-control study by Christy et al. was conducted in 2013 on 120 diabetic patients and found a positive correlation between HbA1c and IDA (6.8+/-1.4%), especially in women (7.02+/-1.58%) [21]. The value of HbA1c was higher in patients with plasma fasting glucose 100-126 mg/dl (7.33+/-1.55%) compared to euglycemic. However, there is a negative correlation between ferritin and hemoglobin. So, the author suggests the correction of IDA in a patient with impaired glycaemic control.

Some studies tried to delineate the impact of Hb level on HbA1c. One such study conducted by Silva et al. showed among 122 participants (67 with anemia, 67 without anemia) has found that the HbA1c level does not vary significantly with the measuring technique (like high-performance liquid chromatography (HPLC) method and immunoturbidimetry method; P=0.192) [19]. Mild anemia has little impact on the HbA1c level, whereas moderate to severe anemia can increase the level of HbA1c. Hence, in people with mild anemia, HbA1c can be used as a diagnostic tool for diabetes. Others have tried to study the impact of IDA on the HbA1c level in particular settings. A study conducted by Inada and Koga in 2017 compared the HbA1c level in gastrectomized subjects with and without IDA [17]. HbA1c level showed a negative correlation with all the indicators of iron deficiency like ferritin, mean corpuscular haemoglobin (MCH), and haemoglobin. Thus, the HbA1c level is higher in gastrectomized subjects with IDA than non-IDA (P=0.003).

Guo et al. in 2019 highlights the need for further studies as there is no clear cut demarcation of effects of IDA on HbA1c level; the cause being multifactorial and the effect being multidimensional [6]. Moreover, due to the lack of standard calibration method to measure HbA1c, other confounding variables like IDA may lead to misdiagnosis or underdiagnosis of diabetes. Also, a review by Naquash and Bader in his article mention that the HbA1c level is dependent on erythrocyte turnover so, in the IDA, HbA1c level increased and in other forms of anemia, HbA1c level is lowered [23]. Furthermore, other studies contradicted the finding. However, the clinical data is insufficient, and further studies are mandatory in delineating the effect of erythrocyte indices in the HbA1c level.

Similarly, a systematic review by English et al. in 2015 concludes that erythrocyte indices are essential confounders in the analysis of HbA1c in patients with anemia and diabetes [7]. So, the author highlighted the need for further study in this regard. In a review by Weykamp in 2013 [5], in anemia due to chronic disease, HbA1c is low due to decreased RBC survival, but in IDA, it is spuriously high, which is assumed to be enticed to altered glycation rate. A similar recommendation by Ahmad and Raft in 2013 suggested more research is needed to know the mechanism by which Hb and HbA1c are related precisely [24]. Furthermore, the author also recommends people who are in the diagnostic threshold to diagnose diabetes in anemia should have another method of diagnostic modality to confirm the disease.

In a nutshell, most of the studies showed a relationship between anemia and the HbA1c level; however, it fails to demonstrate the effect and the goal of optimal HbA1c control in diabetes and non-diabetes.

Study limitations

Data might not have been consistent as we included studies from all around the world. Also, we could not do the systematic review and meta-analysis of all the articles which would strengthen our paper. Furthermore, since we could review only the articles published in the English language, we might have missed the valuable findings observed in the articles published in the local languages. Besides, we reviewed the articles published since 2011 only which could have excluded the important conclusions from the articles published earlier.

## Conclusions

Our study indicates the need for screening for the anemia in patients before commencing the treatment of diabetes diagnosed via the HbA1c level. Furthermore, anemia should be corrected before setting the treatment goal of optimal HbA1c control, especially when the level is in the diagnostic threshold. Also, the purpose of strict HbA1c control is not recommended in the anemic patient before it is corrected. However, further large-scale interventional studies are needed to know precisely, the goal of optimal HbA1c control in diabetic and non-diabetic individuals.
